# Effectiveness of sequential intravenous-to-oral antibiotic switch therapy in hospitalized patients with gram-positive infection: the SEQUENCE cohort study

**DOI:** 10.1007/s10096-016-2661-5

**Published:** 2016-05-14

**Authors:** D. Rodriguez-Pardo, C. Pigrau, D. Campany, V. Diaz-Brito, L. Morata, I. C. de Diego, L. Sorlí, S. Iftimie, R. Pérez-Vidal, G. García-Pardo, T. Larrainzar-Coghen, B. Almirante

**Affiliations:** Department of Infectious Diseases, Hospital Universitari Vall d’Hebron, Universitat Autònoma de Barcelona, Barcelona, Spain; Pharmacy Department, Hospital Universitari Vall d’Hebron, Barcelona, Spain; Department of Internal Medicine, Parc Sanitari Sant Joan de Déu, Sant Boi de Llobregat, Spain; Department of Infectious Diseases, Hosp. Clínic i Provincial, IDIBAPS, Barcelona, Spain; Department of Infectious Diseases, Hospital Universitari Arnau de Vilanova, Lleida, Spain; Department of Infectious Diseases, Hospital del Mar, IMIM, CEXS-UPF, Barcelona, Spain; Department of Internal Medicine, Hospital Universitari Sant Joan de Reus, Reus, Spain; Department of Internal Medicine, Fundació Althaia, Hospital de Sant Joan de Déu, Manresa, Spain; Department of Internal Medicine, Hospìtal Universitari Joan XXIII, IISPV, Universitat Rovira i Virgili, Tarragona, Spain

## Abstract

Switching from intravenous to oral antibiotic therapy may improve inpatient management and reduce hospital stays and the complications of intravenous treatment. We aimed to assess the effectiveness of intravenous-to-oral antibiotic switch therapy and an early discharge algorithm in hospitalized patients with gram-positive infection. We performed a prospective cohort study with a retrospective comparison cohort, recruited from eight tertiary, acute-care Spanish referral hospitals. All patients included had culture-confirmed methicillin-resistant gram-positive infection, or methicillin-susceptible gram-positive infection and beta-lactam allergy and had received intravenous treatment with glycopeptides, lipopeptides, or linezolid. The study comprised two cohorts: the prospective cohort to assess the effectiveness of a sequential intravenous-to-oral antibiotic switch algorithm and early discharge, and a retrospective cohort in which the algorithm had not been applied, used as the comparator. A total of 247 evaluable patients were included; 115 in the prospective and 132 in the retrospective cohort. Forty-five retrospective patients (34 %) were not changed to oral antibiotics, and 87 (66 %) were changed to oral antibiotics without following the proposed algorithm. The duration of hospitalization was significantly shorter in the prospective cohort compared to the retrospective group that did not switch to oral drugs (16.7 ± 18.7 vs 23 ± 13.4 days,* P*  < 0.001). No differences were observed regarding the incidence of catheter-related bacteraemia (4.4 % vs 2.6 %, *P* = 0.621). Our results suggest that an intravenous-to-oral antibiotic switch strategy is effective for reducing the length of hospital stay in selected hospitalized patients with gram-positive infection.

## Introduction

The rising incidence of infections caused by methicillin-resistant gram-positive microorganisms has increased the use of glycopeptide and lipopeptide antibiotics, and linezolid [1–7]. However, glycopeptides and lipopeptides have the disadvantage of requiring intravenous (IV) administration, which often involves prolonged hospitalization and carries a risk of catheter-related complications.

Several studies have demonstrated the efficacy and safety of switching from intravenous to oral antibiotics in clinically stable patients [8–19]. Intravenous-to-oral switch therapy facilitates hospital discharge while maintaining equivalent outcomes; therefore, it can be an effective approach to improve inpatient management and reduce the risk and cost associated with prolonged hospital stays and catheter-related adverse events [9, 14, 16, 17, 20]. Other potential benefits include increased patient satisfaction, and a reduction in potential reservoirs of methicillin-resistant gram-positive microorganisms in the hospital, thereby lowering the likelihood of transmission to uncolonized patients and medical staff. However, although sequential intravenous-to-oral therapy is becoming an increasingly more widely used strategy, many patients still remain hospitalized under IV treatment until their infection resolves.

Proper patient selection, patient health education, and an active therapeutic approach by multidisciplinary medical teams are the key factors leading to success in conversion from intravenous to oral antimicrobials [9, 21]. Daily patient evaluation by an infectious disease specialist has a significant impact on antimicrobial use, facilitating intravenous-to-oral switch and identifying patients who may be suitable for early discharge [22].

The objective of this study was to assess the effectiveness of actively applying an algorithm for intravenous-to-oral antibiotic switch therapy and early discharge in the management of hospitalized patients with gram-positive infections requiring treatment with glycopeptides, lipopeptides, or linezolid.

## Material and methods

This is a prospective cohort study with a retrospective historical comparison cohort, recruited from eight Spanish tertiary referral hospitals. The study includes hospitalized adult patients (older than 17 years) with a culture-confirmed diagnosis of methicillin-resistant gram-positive infection, or methicillin-susceptible gram-positive infection and beta-lactam allergy, who received IV treatment with glycopeptides, lipopeptides, or linezolid. The primary endpoint was the reduction in the length of hospital stay from the time point when gram-positive infection had been diagnosed.

### Study design and population

The study comprised two cohorts: a retrospective cohort (July 2010 to December 2011) in which the switch therapy algorithm was not applied, used as the comparison group, and a prospective cohort to assess the effectiveness of the proposed algorithm over a 1-month follow-up once antimicrobial treatment had been completed (January 2012 to September 2014). Physicians trained in antibiotic management (infectious disease or internal medicine staff) evaluated all patients included in both cohorts.

In the retrospective comparison group, pharmacy records were used to identify patients who had been prescribed IV treatment with glycopeptides, lipopeptides, or linezolid for more than 72 h. The patients were evaluated by the researchers at each centre, and those with a culture-confirmed diagnosis of gram-positive infection who met all the inclusion and none of the exclusion criteria were enrolled in the study.

**Fig. 1:**
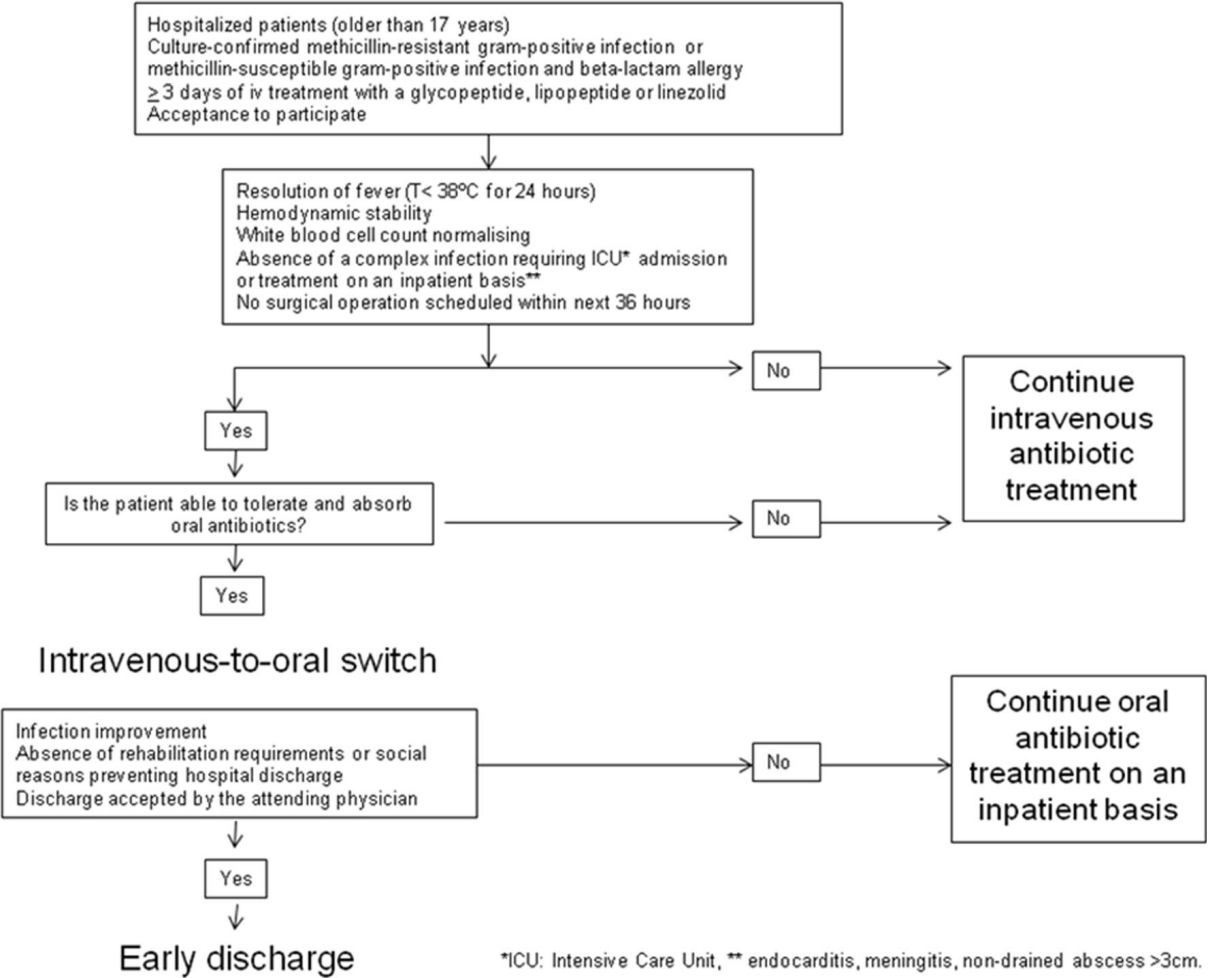
Algorithm for intravenous-to-oral switch therapy and early discharge

In the prospective cohort, daily microbiology and/or pharmacy antibiotic reports were used to identify potential candidates for inclusion among hospitalized patients with a confirmed gram-positive infection. The patient’s suitability for enrolment was formally assessed by using an algorithm designed for intravenous-to-oral antibiotic switch therapy and early discharge (Fig. 1). The inclusion criteria for changing from intravenous-to-oral were improvements in the infection, resolution of fever, hemodynamic stability (systolic blood pressure of ≥ 100 mmHg and no unexplained tachycardia defined as heart rate greater than 100 beats per minute in last 12 h), progressive reductions in white blood cell count, and absence of a complex infection requiring treatment on an inpatient basis (e.g., meningitis, endocarditis). The exclusion criteria were age younger than 18 years, intensive care unit admission at the time of the first evaluation, presence of a non-drained abscess of more than 3 cm, reasonable concerns about potential patient compliance, patient unable to take oral therapy, and refusal to participate. Patients who met all these conditions and signed the informed consent to participate were included in the prospective cohort study. All patients included in the prospective cohort were evaluated according to the following schedule of visits: an initial screening visit (to recruit the patient), daily monitoring visits during hospitalization (first to assess the intravenous-to-oral switch and thereafter to assess the possibility of discharge), and a final study visit at 1 month after the antibiotic schedule had been completed. In addition to the study visits by the researchers, all prospective patients were visited by their attending physicians, who conducted clinical monitoring for as long as was considered necessary to confirm resolution of the patient’s condition. The SEQUENCE study was approved by the Hospital Universitari Vall d’Hebron Institutional Review Board (code HVH-ATB-2010-01).

### Study variables and data collection

The demographic, clinical, laboratory, and microbiological data and the patient’s clinical course and outcome were recorded retrospectively in the retrospective cohort and prospectively in the prospective one. The incidence of catheter-related infection was recorded in both cohorts. All data were anonymously entered into a dedicated database for analysis.

### Definitions

Early intravenous-to-oral antibiotic switch therapy was defined as a switch occurring within 5 days after the start of IV therapy. We chose a 5-day interval, considering that it was the period of time necessary to have all the microbiological data, including, in the case of bacteraemia, the result of control blood cultures obtained within 72 h after starting directed antibiotic treatment. Failure of the proposed algorithm was established on infection-related patient death, need for readmission due to the infectious process, or need to change back to IV treatment because the patient showed no improvement. Early hospital discharge was defined as discharge within 48 h after the switch.

### Statistical analysis

Continuous variables were compared using the Mann–Whitney *U* test, and are expressed as the mean and standard deviation (SD) or the median and interquartile range (IQR). Categorical variables were compared using the chi-square test or Fisher's exact test. Data were analysed using SPSS (Statistical Package for the Social Sciences) software (version 18.0). All analyses were 2-tailed, and a *P* value <0.05 was considered statistically significant.

## Results

**Fig. 2:**
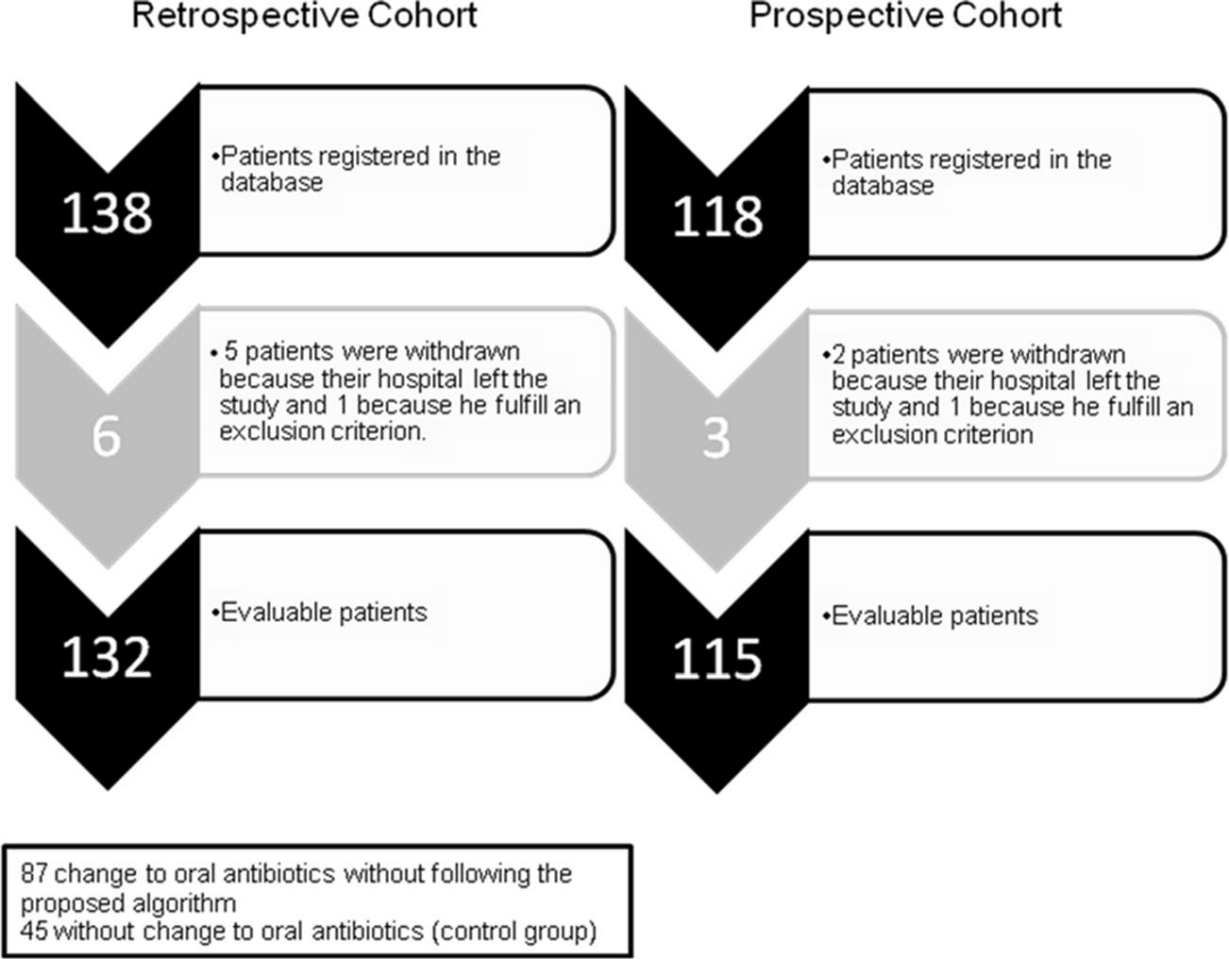
Patients included in the study

A total of 247 evaluable patients, 142 (57.5 %) men, with a median age of 70 years (57–80) were included: 132 patients were in the retrospective group, which comprised 45 (34 %) who did not switch to oral antibiotics and 87 (66 %) who switched without following the proposed algorithm, and 115 patients were in the prospective cohort (Fig. 2). Patients in the retrospective cohort who had been converted to oral antibiotic treatment without following the proposed algorithm were mainly affected with osteoarticular infection (32 of 34 cases, 94 %), skin and soft tissue infection (16 of 22, 73 %), respiratory infection (nine of 14, 65 %), and urinary infection (12 of 20, 60 %). The change to oral therapy was used the least in patients with catheter-related infection (seven of 22, 32 %). Length of hospital stay from the diagnosis of gram-positive infection was shorter in patients receiving oral continuation therapy than in those not receiving it (16.9 ± 11.0 days vs 30.6 ± 53.0; *P* = 0.003).

The prospective patients (*N* = 115) were compared with the patients in the retrospective cohort who did not change to oral antibiotics (*N* = 45) (Table 1). The two groups were comparable for age (66.1 ± 16.2 vs 69.2 ± 16.9 years, *P* = 0.221), sex (male, 55.7 % vs 60.0 %, *P* = 0.723), and hospitalization requirement in the previous 6 months (61, 53.0 % vs 21, 46.7 %, *P* = 0.487). The retrospective cohort included a higher percentage of bloodstream infections (26, 57.8 % vs 16, 17.6 %, *P* < 0.001) and catheter-related infections (15, 33.3 % vs 5, 4.3 %, *P* < 0.001), but fewer osteoarticular infections (2, 4.4 % vs 71, 61.7 %, *P* < 0.001). With regard to catheter-related infections, four of the five prospective patients with a catheter-related infection were successfully changed to oral linezolid. Intravenous antibiotic was administered for a longer period in retrospective patients than in prospective ones (mean duration of IV antibiotics 14 ± 9.3 vs 7.8 ± 4.5 days, *P* < 0.001). All patients in the prospective group were switched to oral continuation antimicrobial therapy with one or more of the following that antibiotics: linezolid 63 (55 %), cotrimoxazole 38 (33 %), levofloxacin 14 (12 %), and clindamycin 11 (9.5 %). In 48 patients (42 %) with an osteoarticular infection, rifampin was co-administered as a part of their oral antibiotic regimen.

**Table 1:** Comparison between patients in the retrospective cohort who did not change to oral antibiotics and patients in the prospective cohort

	Retrospective cohort *n* = 45	Prospective cohort *n* = 115	*P*
Age, years, mean (SD)	69.2 (16.9)	66.1 (16.2)	0.221
Sex, male	27 (60)	64 (55.7)	0.723
Patients hospitalized in previous 6 months	21 (46.7)	61 (53.0)	0.487
Site of infection			
Catheter-related infections	15 (33.3)	5 (4.3)	<0.001
Urinary tract infections	8 (17.8)	8 (7)	<0.074
Skin and soft-tissue infections	6 (13.3)	21 (18.3)	0.639
Respiratory infections	5 (11.1)	4 (3.5)	0.118
Osteoarticular infections^a^	2 (4.4)	71 (61.7)	<0.001
Abscess	0	3 (1.9)	0.001
Other infections^b^	9 (20)	3 (2.6)	0.560
Positive blood culture	26 (57.8)	16 (13.9)	<0.001
Microbiological aetiology^c^			
Methicillin-resistant *Staphylococcus aureus*	15 (33.3)	54 (47)	0.118
Methicillin-susceptible *Staphylococcus aureus* ^d^	0	4 (3.5)	0.577
Methicillin-resistant coagulase-negative staphylococci	14 (31.1)	44 (38.3)	0.398
Methicillin-susceptible coagulase-negative staphylococci^d^	3 (6.7)	4 (3.5)	0.375
Ampicillin-resistant *Enterococcus faecium*	11 (24.4)	10 (8.7)	0.008
Ampicillin-susceptible *Enterococcus spp.* ^d^	1 (2.2)	3 (2.6)	0.888
*Streptococcus pneumoniae* ^d^	1 (2.2)	0	0.281
Length of IV antibiotics, days, mean (SD)	14.2 (9.3)	7.8 (4.5)	<0.001
Length of hospital stay, days, mean (SD)^e^	23 (13.4)	16.7 (18.7)	<0.001
Treatment catheter-related infection	2 (4.4)	3 (2.6)	0.621
Related mortality	1 (2.2)	1 (0.9)	>0.999

Values are expressed as n (%) unless otherwise indicated

^a^63 of 73 osteoarticular infections were prosthetic joint infections or osteosynthesis infections, and ten osteoarticular infections were chronic osteomyelitis

^b^Other infections include five primary bacteraemia, three peritonitis, two cholangitis, one empyema and one pacemaker-associated infection

^c^Four patients included in the prospective cohort had polymicrobial infection due to two different gram-positive isolates. Therefore, 119 isolates were identified in the prospective cohort

^d^In patients with beta-lactam allergy

^e^Length of hospital stay from diagnosis of gram-positive infection to hospital discharge or death

### Outcome analysis

Only 59 prospective patients (51 %) were afforded an early discharge. Nonetheless, there was reduction in the length of hospital stay in prospective patients compared to the retrospective cohort (16.7 ± 18.7 vs 23.0 ± 13.4 days, *P* < 0.001). Only five episodes of catheter-related infection related to the treatment were detected, with no differences between the two cohorts (3, 2.6 % vs 2, 4.4 % *P* = 0.621). The proposed switch algorithm failed in ten prospective patients (8.7 %). Eight patients required readmission due to failure to improve in seven patients and a related adverse effect (*Clostridium difficile* infection) in one patient (Table 2). One patient who had been switched to oral rifampin plus linezolid required a change back to IV antibiotic due to gastrointestinal intolerance, and one patient with a methicillin-resistant *S. aureus* respiratory infection died due to progression of the infection and respiratory failure. One patient in the retrospective cohort also died because of progression of the infection.

**Table 2:** Patients in the prospective cohort who required hospital readmission due to the infection and changed back to IV antibiotic treatment

N	Type of infection	Causative microorganisms	Initial IV antibiotic	Oral antibiotic	Reason for readmission	Comments
1	Knee PJI	CONS	DAP	CLIN+RF	*C. difficile-*associated diarrhoea	Antibiotics were not reintroduced
2	Cellulitis	MRSA, GNB	VAN+CIP	COT+ CIP	Persistence of infection	Surgical debridement
3	Knee PJI^a^	CONS	VAN	LIN	Persistence of PJI	2-stage removal
4	Chronic osteomyelitis	CONS	TEICO	LIN+RF	Persistence of infection	New surgery
5	Knee PJI^a^	MRSA	DAP	LIN+RF	Persistence of PJI	2-stage removal
6	Shoulder PJI^a^	CONS	VAN	COT+ RF	Persistence of PJI	2-stage removal
7	Chronic osteomyelitis	CONS	TEICO	LIN	Persistence of infection+GNB superinfection	New surgery
8	Knee PJI	CONS	DAP	LIN	Persistence of infection	New surgical approach

*PJI* prosthetic joint infection, *CONS* coagulase-negative staphylococci, *MRSA* methicillin-resistant *S. aureus*, *GNB* gram-negative bacilli, *MSSA* methicillin-susceptible *S. aureus* (in patients with beta lactam allergy), *DAP* daptomycin, *CLIN* clindamycin, *RF* rifampicin, *VAN* vancomycin, *CIP* Ciprofloxacin, *COT* Cotrimoxazole, *LIN* linezolid, *TEICO* teicoplanin

^a^Three patients diagnosed with PJI had been previously treated with debridement, antibiotics, and implant retention, but this strategy failed and all of them required prosthesis removal using a two-stage approach

Of particular note, 32 of the 34 patients with osteoarticular infection in the retrospective cohort had been switched to oral antibiotics without following the proposed algorithm, and for that reason, were excluded from the main comparative analysis. Therefore, we performed two different sub-analyses to enable comparability between the two cohorts. First, we analysed the length of hospital stay excluding all patients with osteoarticular infection in both cohorts, which left 43 retrospective patients and 44 prospective ones for the comparative analysis. The reduction in hospital stay in the prospective cohort compared with the retrospective one was confirmed (12.5 ± 9.5 vs 21.8 ± 18.1 days, *P* < 0.001). Second, we compared all patients with osteoarticular infections sequenced to oral therapy in the two cohorts (32 retrospective and 71 prospective patients). The time interval from diagnosis of infection to the switch to oral antibiotics was lower in prospective patients (9.2 ± 8.0 vs 12.2 ± 10.1 days, *P* = 0.006), although the length of hospitalization did not significantly differ (19.2 ± 13.9 vs 21.2 ± 16.3 days, *P* = 0.183). Only 35 patients (50 %) were afforded an early discharge. The reasons preventing an early discharge were rehabilitation requirements in 15 patients, social reasons (mainly long-term care facility requirement) in 14 patients, refusal to discharge by the attending physician in four patients, and uncontrolled co-morbidity in two patients.

## Discussion

The results of this study confirm the effectiveness of an algorithm of intravenous-to-oral antibiotic switch therapy in terms of reducing the length of hospitalization and duration of intravenous antibiotic therapy in all patient according to all the diagnoses included, with the exception of those with osteoarticular infection, in whom the length of hospitalization was comparable in those switching or not.

More than 10 years ago, it was reported that the length of hospital stay and the duration of IV antibiotic treatment can be shortened when an early switch policy is introduced once culture results are known [23]. Nonetheless, few studies to date have investigated the effectiveness of an intravenous-to-oral antibiotic switch algorithm. In one randomized study, 50 % of patients initially treated with parenteral antibiotics had their regimens refined after 3 days of therapy, and these modifications resulted in good clinical outcomes with a substantial reduction in antibiotic expenditure [8]. Another study identified patients likely to be suitable for early discharge as those with skin or soft-tissue infection, no high-risk comorbidities, and less than five other regularly prescribed drugs [9]. It has been also demonstrated that the intervention of an antimicrobial management team results in earlier intravenous-to-oral switching and shorter duration of antibiotic therapy, with the potential for early discharge in some of the patients [22]. Our results are consistent with the findings in these studies.

Even though a reduction in the length of hospital stay was seen in our series, only 50 % of patients were afforded early discharge. This indicates that other factors in addition to the treatment administration route can delay discharge, the most common ones in our series being social and rehabilitation requirements.

It is noteworthy that most patients with a catheter-related infection in the retrospective cohort had not been switched to an oral antibiotic, and that only five prospective patients had catheter-related infection. Although intravenous-to-oral antibiotic switch therapy is not an established practice in catheter-related infection, a multicentre comparative study showed that linezolid is as effective as standard vancomycin therapy when treating catheter-related bloodstream infections caused by gram-positive microorganisms [24]. In our series, four of five catheter-related infections were safely switched to oral linezolid once bacteraemia had resolved. This is a small number of patients, but Wilcox et al. had just published their multicentre comparative study [24]. Probably because their results were still little-known, intravenous-to-oral antibiotic switch was uncommon in patients with catheter-related bacteraemia. However, based on Wilcox’s results and our experience, we consider that patients with an uncomplicated catheter-related gram-positive bacteraemia are a possible target group for effective oral switch therapy with linezolid.

With regard to orthopaedic infection, intravenous-to-oral antibiotic switch therapy is established practice in our setting, as 32 of 34 retrospective patients had been sequenced spontaneously by their attending physician without following any algorithm. Application of an intravenous-to-oral switch algorithm by a specialist enabled earlier switching, but it did not significantly shorten hospital stay in this subgroup of patients. Factors such as surgical wound healing and the need for functional rehabilitation are likely the main reasons for continued hospitalization.

Seven prospective patients who had been switched to oral antibiotics required hospital readmission and a new intravenous treatment with antibiotics. All these patients (six osteoarticular infections and one complicated skin infection) required surgical debridement to resolve the infection, thus illustrating the importance of an extensive surgical approach in these conditions.

Finally, we would like to draw your attention to one last issue. One theoretical benefit of intravenous-to-oral antibiotic switch therapy is that it can reduce catheter-related adverse events [20], but we found no differences between prospective and retrospective patients in the incidence of catheter-related bacteraemia. Catheter-associated complications are often underreported in medical charts, and this may partially explain the low incidence of catheter-related bacteraemia in retrospective patients. This can be considered a limitation of our study.

Other possible limitations are related to the methods: we enrolled patients with various infections caused by different microorganisms which can make comparisons difficult, we compared a prospective group with a retrospective one in which certain data were difficult to obtain, and there was some lack of uniformity between the eight centres included in the study. However, it had been necessary to include a large number of patients to perform subgroup analysis, and comparison with a prospective group in which there were no intervention with respect to antibiotic treatment could lead to a conflict of ethics in a situation that is considered beneficial for the patient. The lack of uniformity can also have a positive side, as it shows that the intravenous-to-oral algorithm can be effectively used in a variety of clinical settings once candidate patients have been carefully selected using a systematic approach with criteria established by consensus.

In conclusion, our data suggest that intravenous-to-oral antibiotic switch therapy is a useful approach for managing selected hospitalized patients with gram-positive infection, as it safely reduces the length of hospital stay and intravenous treatment. In patients with osteoarticular infection, the timing of the switch to oral antibiotics was earlier when our strategy was implemented, but the length of hospital stay did not significantly differ, probably because other factors, such as surgical wound healing and rehabilitation requirements, prevent early discharge.

## Appendix A

### List of collaborators

This study was possible thanks to the work of the following collaborators: Albert Pahissa and Roger Sorde (Hospital Universitari Vall d’Hebron, Barcelona); Elisabeth Rovira (Parc Sanitari Sant Joan de Déu, Sant Boi de Llobregat); Alex Soriano (Hosp. Clínic i Provincial, IDIBAPS, Barcelona); Alfonso Tapiz Reula (Fundació Althaia, Hospital de Sant Joan de Déu, Manresa); Laura Canadell Vilarrasa and Josepa Tapiol Oliva (Hospìtal Universitari Joan XXIII, IISPV, Universitat Rovira i Virgili, Tarragona); F. Barcenilla (Hospital Universitari Arnau de Vilanova, Lleida).

## Abbreviations

IV: intravenous
SD: standard deviation
IQR: interquartile range
